# Supplementary material for: CRISPR knockout genome-wide screens identify the HELQ-RAD52 axis in regulating the repair of cisplatin-induced single-stranded DNA gaps
Source: Nucleic Acids Res. 2024 Nov 12;52(22):13832–48. doi: 10.1093/nar/gkae998 (PMC11662931; doi:10.1093/nar/gkae998)
Supplement: gkae998_Supplemental_Files [file gkae998_supplemental_files.zip › Supplementary Material.pdf]

SUPPLEMENTARY MATERIAL

Legends to Supplementary Tables

Supplementary Table S1. MAGeCK analyses of the CRISPR screens identifying genes whose loss confers increased sensitivity to cisplatin in HeLa-EV cells.

Supplementary Table S2. MAGeCK analyses of the CRISPR screens identifying genes whose loss confers increased sensitivity to cisplatin in HeLa PRIMPOL-overexpressing cells.

Supplementary Table S3. MAGeCK analyses of the CRISPR screens identifying genes whose loss confers increased sensitivity to cisplatin in U2OS-EV cells.

Supplementary Table S4. MAGeCK analyses of the CRISPR screens identifying genes whose loss confers increased sensitivity to cisplatin in U2OS PRIMPOL-overexpressing cells.

Supplementary Table S5. The source data underlying each of the main and supplementary figure panels, including: the values plotted in graphs, the exact p-values, and the uncropped blots.

Supplementary Figure S1

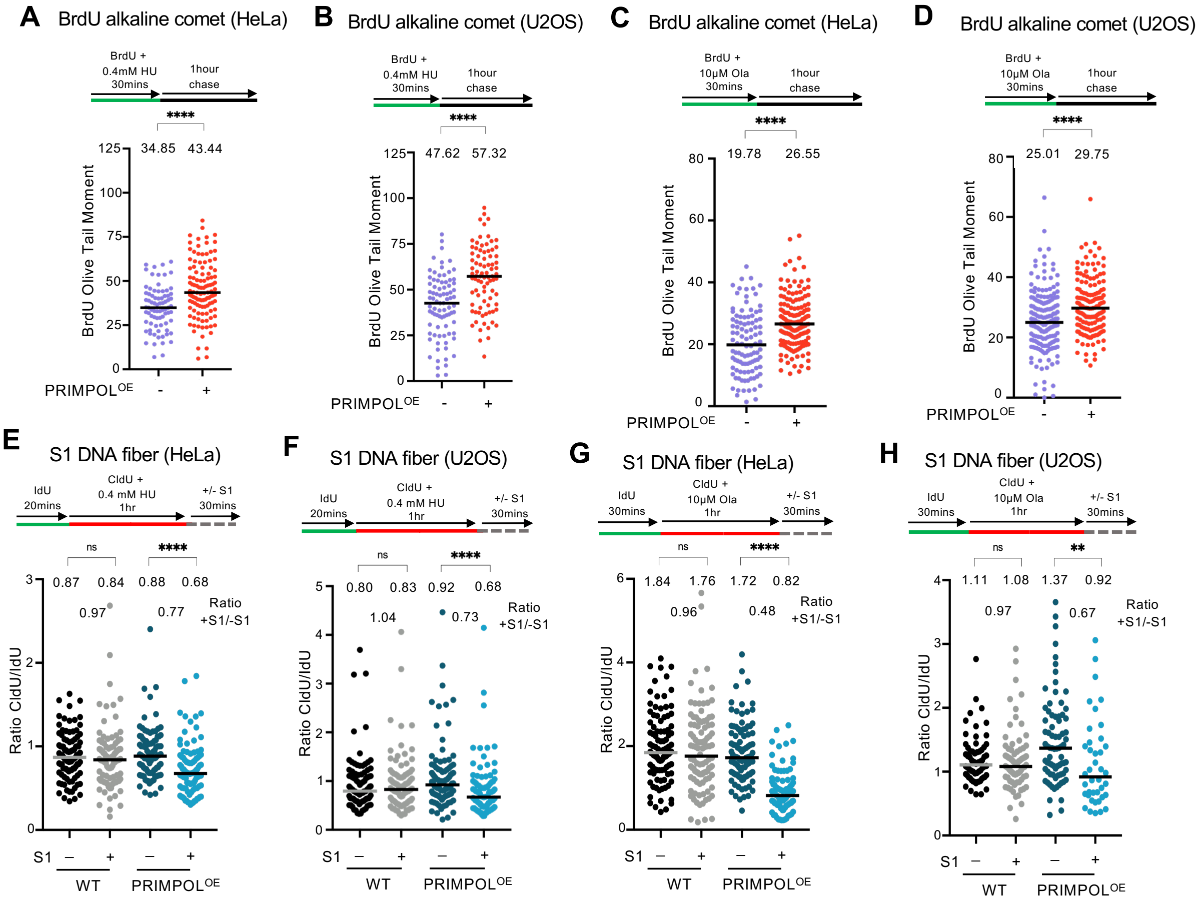

Supplementary Figure S1. Induction of ssDNA gaps in PRIMPOL-overexpressing cells. A-D. BrdU alkaline comet assays showing that PRIMPOL overexpression in HeLa (**A,C**) and U2OS (**B,D**) cells causes accumulation of replication-associated ssDNA gaps upon treatment with 0.4mM HU (**A,B**) or 10μM olaparib (**C,D**). At least 75 nuclei were quantified for each condition. The median values are marked on the graph and listed at the top. Asterisks indicate statistical significance (Mann-Whitney, two-tailed). Schematic representations of the assay conditions are shown at the top.

E-G. S1 nuclease DNA fiber combing assays showing that PRIMPOL overexpression in HeLa (**E,G**) and U2OS (**F,H**) cells causes accumulation of nascent strand ssDNA gaps upon treatment with 0.4mM HU (**E,F**) or 10μM olaparib (**G,H**). The ratio of CldU to IdU tract lengths is presented, with the median values marked on the graphs and listed at the top. At least 40 tracts were quantified for each sample. Asterisks indicate statistical significance (Mann-Whitney, two-tailed). Schematic representations of the assay conditions are shown at the top.

Supplementary Figure S2

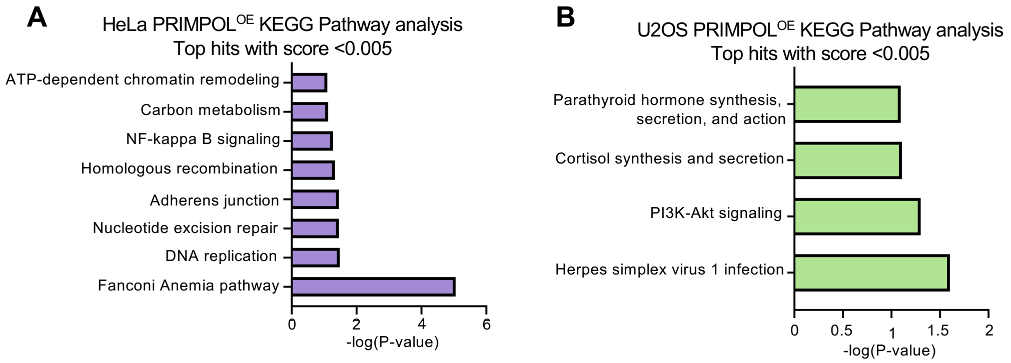

C

Gene	Rank Cisplatin sensitivity HeLa PRIMPOL^{OE}	Rank Cisplatin sensitivity U2OS PRIMPOL^{OE}	Function
HELQ	174	191	ssDNA ATPase and DNA helicase
GDI1	166	6	GDP dissociation inhibitor in GDP-GTP exchange reaction
THNSL2	211	16	Threonine synthase-like protein with phospho-lyase activity
OR10W1	140	68	Olfactory receptor protein
GPN1	304	132	Guanosine triphosphatase enzyme that may play a role in DNA repair
STAB1	188	169	Transmembrane receptor with potential functions in angiogenesis, lymphocyte homing, cell adhesion
PRDX2	111	212	Antioxidant enzyme
ZFP62	169	246	Zinc finger protein with DNA-binding transcription activator activity
SLC35E2	159	257	Solute carrier predicted to be involved in transmembrane transport
OR8J3	285	279	Olfactory receptor protein
DIRAS2	84	324	Ras monomeric GTPase

Supplementary Figure S2. Analyses of CRISPR screens to identify genes causing cisplatin sensitivity to PRIMPOL-overexpressing cells.

A,B. Biological pathway analyses using KEGG analyses of the top hits with MAGeCK score lower than 0.005 which cause cisplatin sensitivity in PRIMPOL-overexpressing HeLa (**A**) and U2OS (**B**) cells. KEGG terms with negative logP greater than 1.08 are presented.

C. Table showing the common hits (MAGeCK score lower than 0.005) in the HeLa and U2OS PRIMPOL-overexpressing cisplatin sensitivity screens, their ranks and their biological functions.

Supplementary Figure S3

A

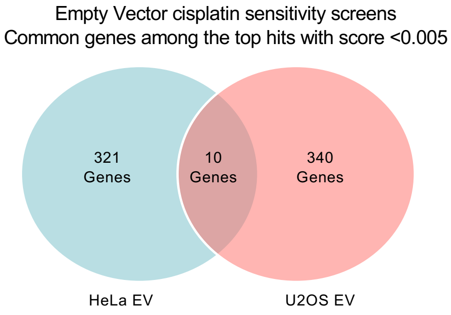

C

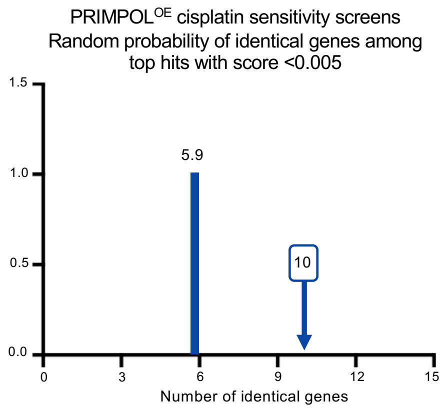

B

Gene	Rank Cisplatin sensitivity HeLa EV	Rank Cisplatin sensitivity U2OS EV	Function
ATM	51	164	DNA damage checkpoint activation
PAPPA2	90	334	Metzincin metalloproteinase; cleaves insulin growth factor-binding protein 5
BEST2	98	159	Bestrophin family anion channel
SMTNL1	109	21	Striated and smooth muscle contraction
TMEM255B	116	332	Integral membrane component
RTN4RL1	168	303	Potential negative regulation of axon regeneration
DTX3L	215	170	E3 Ubiquitin ligase; STAT protein binding activity
CASC3	250	158	Component of exon junction complex; nonsense-mediated mRNA decay
HAS2	329	77	Hyaluranic acid synthase
FANCA	91	320	DNA repair; Interstrand DNA cross-link repair

Supplementary Figure S3. Analyses of CRISPR screens to identify genes causing cisplatin sensitivity to control (Empty Vector) cells.

A. Diagram showing the overlap of identical genes within the top hits with MAGeCK score lower than 0.005 which cause cisplatin sensitivity in control (Empty Vector) HeLa and U2OS cells.

B. Table showing the common hits (MAGeCK score lower than 0.005) in the HeLa and U2OS control (Empty Vector) cisplatin sensitivity screens, their ranks and their biological functions.

C. The number of common genes within the top hits with MAGeCK score lower than 0.005 which cause cisplatin sensitivity in control (Empty Vector) HeLa and U2OS cells (namely 10) is slightly higher than the random probability of identical hits, which is 5.9.

Supplementary Figure S4

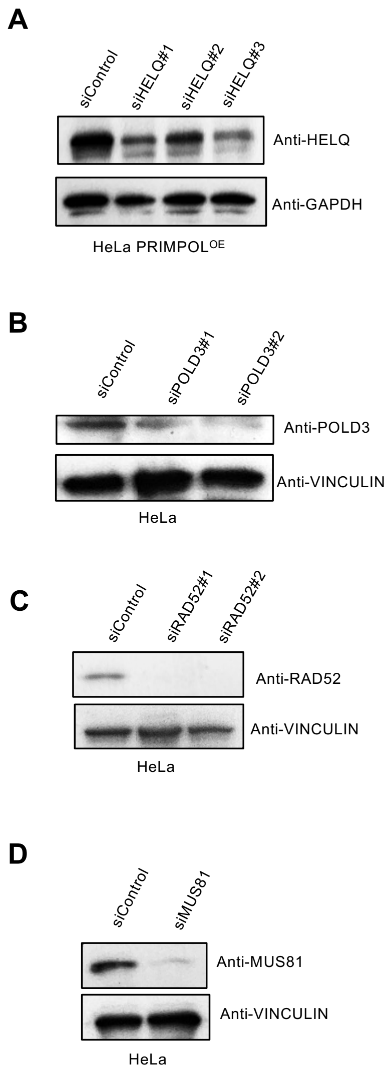

Supplementary Figure S4. Confirmation of gene knockdowns. Western blots showing siRNA-mediated knockdown of HELQ (**A**), POLD3 (**B**), RAD52 (**C**), and MUS81 (**D**) in HeLa cells are presented.

Supplementary Figure S5

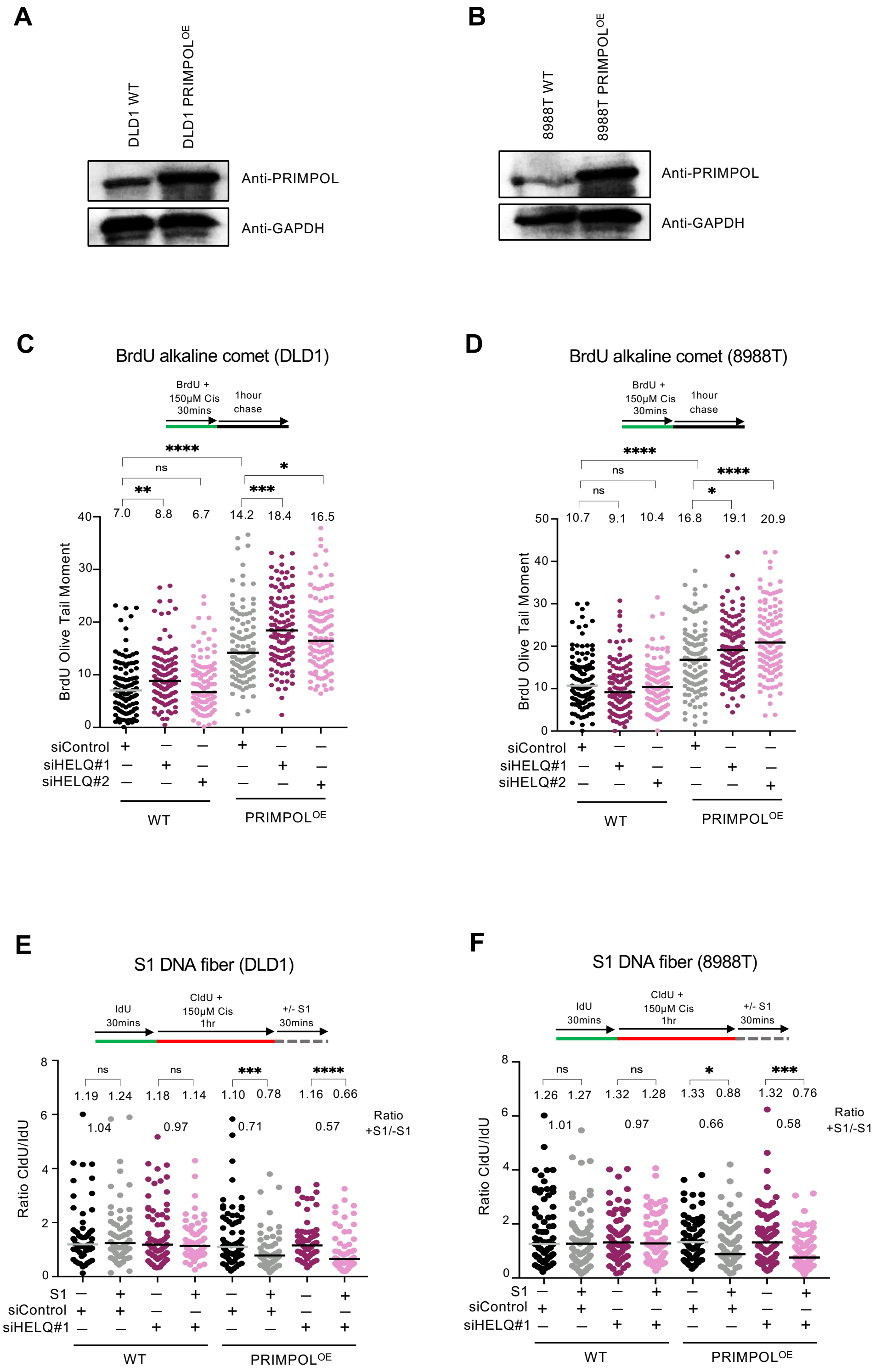

Supplementary Figure S5. PRIMPOL-overexpressing cells accumulate cisplatin-induced ssDNA gaps.

A,B. Western blots showing PRIMPOL overexpression in DLD1 (**A**) and 8988T (**B**) cells.

C,D. BrdU alkaline comet assays showing that PRIMPOL overexpression in DLD1 (**C**) and 8988T (**D**) cells causes accumulation of replication-associated ssDNA gaps upon treatment with 150μM cisplatin. At least 100 nuclei were quantified for each condition. The median values are marked on the graph and listed at the top. Asterisks indicate statistical significance (Mann-Whitney, two-tailed). Schematic representations of the assay conditions are shown at the top.

E,F. S1 nuclease DNA fiber combing assays showing that PRIMPOL overexpression in DLD1 (**E**) and 8988T (**F**) cells causes accumulation of nascent strand ssDNA gaps upon treatment with 150μM cisplatin. The ratio of CldU to IdU tract lengths is presented, with the median values marked on the graphs and listed at the top. At least 60 tracts were quantified for each sample. Asterisks indicate statistical significance (Mann-Whitney, two-tailed). Schematic representations of the assay conditions are shown at the top.

Supplementary Figure S6

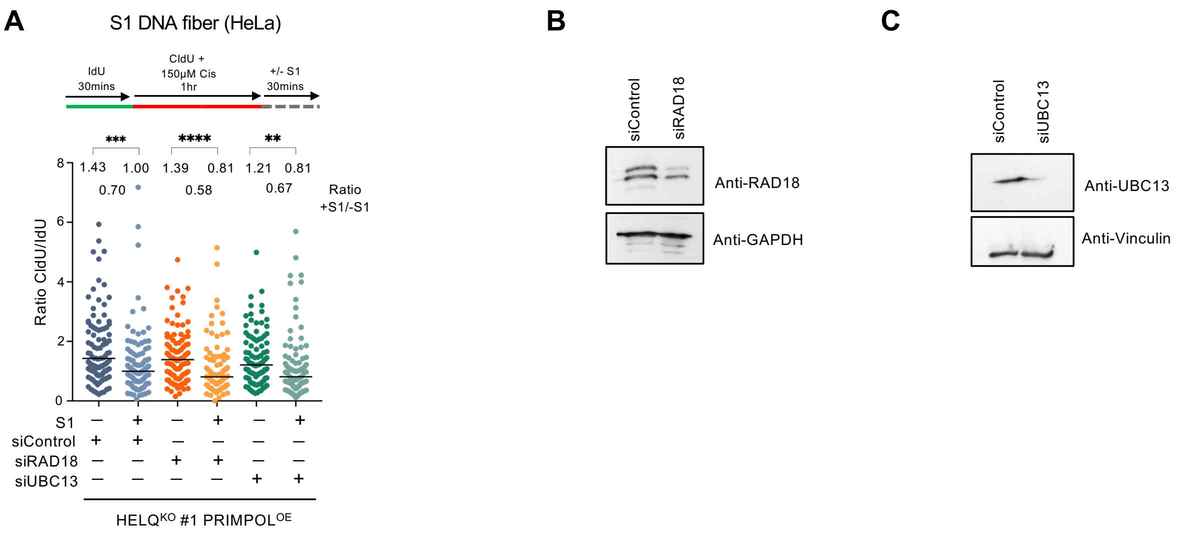

Supplementary Figure S6. Impact of translesion synthesis on PRIMPOL-overexpressing cells.

A. S1 nuclease DNA fiber combing assays in HELQ-knockout PRIMPOL-overexpressing HeLa cells upon treatment with 150μM cisplatin showing the impact of RAD18 or UBC13 depletion. The ratio of CldU to IdU tract lengths is presented, with the median values marked on the graphs and listed at the top. The +S1/-S1 ratios of the median values are also presented. At least 100 tracts were quantified for each sample. Asterisks indicate statistical significance (Mann-Whitney, two-tailed). A schematic representation of the assay conditions is shown at the top.

B,C. Western blots showing siRNA-mediated knockdown of RAD18 (**B**) and UBC13 (**C**).

Supplementary Figure S7

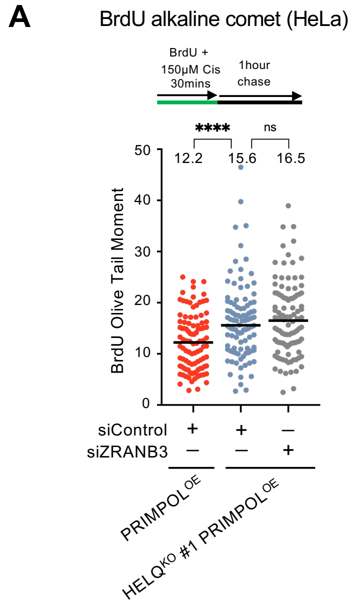

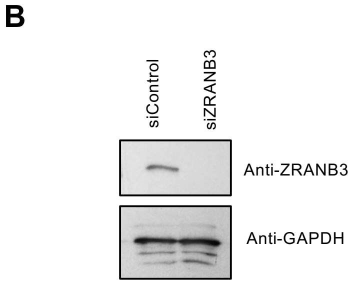

Supplementary Figure S7. Impact of fork reversal on HELQ-knockout PRIMPOL-overexpressing cells

A. BrdU alkaline comet assay in HELQ-knockout PRIMPOL-overexpressing HeLa cells upon treatment with 150μM cisplatin showing no impact of ZRANB3 depletion. At least 100 nuclei were quantified for each condition. The median values are marked on the graph and listed at the top. Asterisks indicate statistical significance (Mann-Whitney, two-tailed). A schematic representation of the assay conditions is shown at the top.

B. Western blots showing siRNA-mediated knockdown of ZRANB3.

Supplementary Figure S8

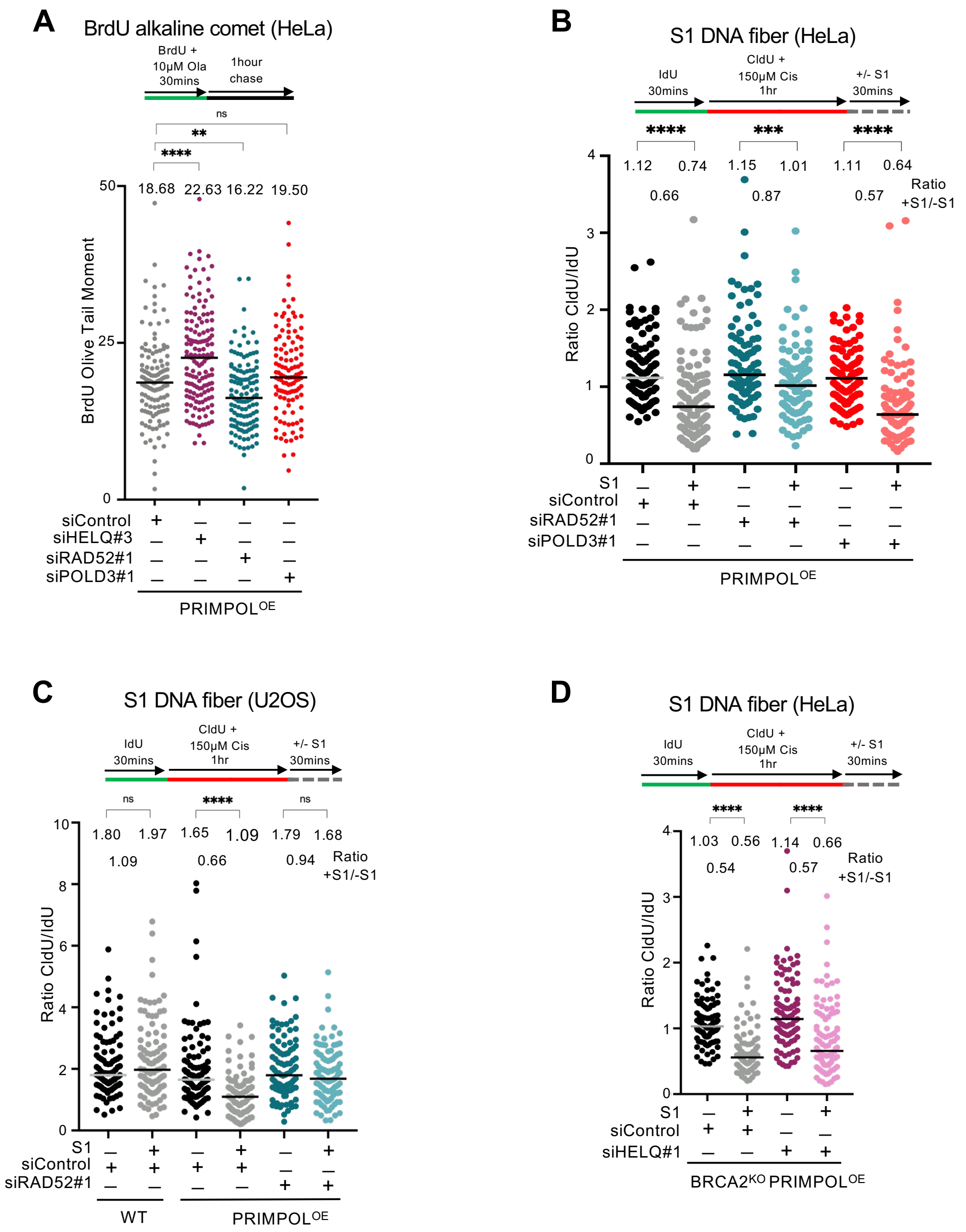

Supplementary Figure S8. Impact of HELQ, RAD52 and POLD3 on ssDNA gap accumulation in PRIMPOL-overexpressing cells.

A. BrdU alkaline comet assays showing that RAD52 depletion reduces ssDNA gap accumulation upon treatment with 10µM olaparib in PRIMPOL-overexpressing HeLa cells, while

POLD3 depletion does not affect it. At least 110 nuclei were quantified for each condition. The median values are marked on the graph and listed at the top. Asterisks indicate statistical significance (Mann-Whitney, two-tailed). A schematic representation of the assay conditions is shown at the top.

B,C. S1 nuclease DNA fiber combing assays showing that RAD52 depletion suppresses ssDNA gap accumulation upon treatment with 150μM cisplatin in PRIMPOL-overexpressing HeLa (**B**) and U2OS (**C**) cells, while POLD3 depletion does not affect it. The ratio of CldU to IdU tract lengths is presented, with the median values marked on the graphs and listed at the top. The +S1/-S1 ratios of the median values are also presented. At least 85 tracts were quantified for each sample. Asterisks indicate statistical significance (Mann-Whitney, two-tailed). Schematic representations of the assay conditions are shown at the top.

D. S1 nuclease DNA fiber combing assays showing that HELQ depletion does not impact ssDNA gap accumulation upon treatment with 150μM cisplatin in PRIMPOL-overexpressing BRCA2-knockout HeLa cells. The ratio of CldU to IdU tract lengths is presented, with the median values marked on the graphs and listed at the top. The +S1/-S1 ratios of the median values are also presented. At least 100 tracts were quantified for each sample. Asterisks indicate statistical significance (Mann-Whitney, two-tailed). A schematic representation of the assay conditions is shown at the top.
